# Differences in Basic Life Support Knowledge Between Junior Medical Students and Lay People: Web-Based Questionnaire Study

**DOI:** 10.2196/25125

**Published:** 2021-02-23

**Authors:** Ludovic Sturny, Simon Regard, Robert Larribau, Marc Niquille, Georges Louis Savoldelli, François Sarasin, Eduardo Schiffer, Laurent Suppan

**Affiliations:** 1 Division of Emergency Medicine Department of Anesthesiology, Clinical Pharmacology, Intensive Care and Emergency Medicine University of Geneva Hospitals and Faculty of Medicine Geneva Switzerland; 2 Division of Anesthesiology Department of Anesthesiology, Clinical Pharmacology, Intensive Care and Emergency Medicine University of Geneva Hospitals and Faculty of Medicine Geneva Switzerland; 3 Unit of Development and Research in Medical Education Faculty of Medicine University of Geneva Geneva Switzerland

**Keywords:** basic life support, cardiopulmonary resuscitation, medical students, undergraduate medical education, out-of-hospital cardiac arrest, life support, cardiopulmonary, medical education

## Abstract

**Background:**

Early cardiopulmonary resuscitation and prompt defibrillation markedly increase the survival rate in the event of out-of-hospital cardiac arrest (OHCA). As future health care professionals, medical students should be trained to efficiently manage an unexpectedly encountered OHCA.

**Objective:**

Our aim was to assess basic life support (BLS) knowledge in junior medical students at the University of Geneva Faculty of Medicine (UGFM) and to compare it with that of the general population.

**Methods:**

Junior UGFM students and lay people who had registered for BLS classes given by a Red Cross–affiliated center were sent invitation links to complete a web-based questionnaire. The primary outcome was the between-group difference in a 10-question score regarding cardiopulmonary resuscitation knowledge. Secondary outcomes were the differences in the rate of correct answers for each individual question, the level of self-assessed confidence in the ability to perform resuscitation, and a 6-question score, “essential BLS knowledge,” which only contains key elements of the chain of survival. Continuous variables were first analyzed using the Student *t* test, then by multivariable linear regression. Fisher exact test was used for between-groups comparison of binary variables.

**Results:**

The mean score was higher in medical students than in lay people for both the 10-question score (mean 5.8, SD 1.7 vs mean 4.2, SD 1.7; *P*<.001) and 6-question score (mean 3.0, SD 1.1 vs mean 2.0, SD 1.0; *P*<.001). Participants who were younger or already trained scored consistently better. Although the phone number of the emergency medical dispatch center was well known in both groups (medical students, 75/80, 94% vs lay people, 51/62, 82%; *P*=.06), most participants were unable to identify the criteria used to recognize OHCA, and almost none were able to correctly reorganize the BLS sequence. Medical students felt more confident than lay people in their ability to perform resuscitation (mean 4.7, SD 2.2 vs mean 3.1, SD 2.1; *P*<.001). Female gender and older age were associated with lower confidence, while participants who had already attended a BLS course prior to taking the questionnaire felt more confident.

**Conclusions:**

Although junior medical students were more knowledgeable than lay people regarding BLS procedures, the proportion of correct answers was low in both groups, and changes in BLS education policy should be considered.

## Introduction

Basic life support (BLS) maneuvers and use of automated external defibrillators (AED) have been shown to greatly increase the survival rate after out-of-hospital cardiac arrests (OHCA) [1]. Nevertheless, their application rate remains very different from one region to another [2,3].

In Switzerland, most regions lack a systematic BLS training program for the general population, even though more than 8000 OHCA occur every year in the country [4]. In Geneva, BLS was provided in less than 40% of OHCA cases between 2009 and 2012 [5].

Medical students might unexpectedly encounter OHCA cases outside of the hospital or university environment and might, given their status, be expected to take care of the situation. Many studies carried out in different medical education systems around the world have however concluded that BLS knowledge among health care students is generally limited [6-10].

Since the first BLS training session for medical students of the University of Geneva – Faculty of Medicine (UGFM) only takes place during the second of their 6-year curriculum, our hypothesis was that they might lack critical knowledge regarding BLS prior to this course. These medical students might, however, be unpredictably faced with OHCA and be expected to respond swiftly and adequately given their chosen profession [11]. Our goal was to assess BLS knowledge among these students prior to their first BLS training and to compare it with that of the general population.

## Methods

### Study Design

A cross-sectional, web-based, questionnaire study compliant with the CHERRIES guidelines was carried out between October 2019 and April 2020 [12]. Such studies do not come within the scope of the Swiss Federal Act on Research involving Human Beings [13]. The study protocol was nevertheless submitted to the regional ethics committee, which declared the project “non-objectionable” (Request #2019-02047).

### Online Platform and Survey Development

An internet platform was developed using the Joomla 3.9 content management system (Open Source Matters, New York, NY). The Community Surveys Pro component version 5 (CoreJoomla, Hyderabad, India) was used to create the questionnaire.

A structured online questionnaire containing 19 questions requiring either open or closed answers was created on the platform (Multimedia Appendix 1). The number of questions was kept below 20 to limit dropout attrition [14-16]. Ten questions were used to assess BLS knowledge. These questions were prepared according to the 2015 International Consensus on Cardiopulmonary Resuscitation and Emergency Cardiovascular Care Science With Treatment Recommendations [17]. The questionnaire was displayed over a total of 4 pages (Table 1). Both the internet platform and the questionnaire were thoroughly tested for usability and user-friendliness by all coauthors before beginning the study.

**Table 1:** Survey structure and questions.

Survey page and field, Question		Type of question
**Introduction: consent**		
	N/A^a^	N/A
**1: demographics**		
	Year of birth	Open (Regex^b^)
	Gender	MCQ^c^
**2: general BLS^d^ knowledge**		
	Ever heard of BLS or ACLS^e^ before	Yes/No
	Meaning of “AED”^f,g^	Open
	Year of the last BLS guidelines update	Open (Regex)
	Phone number of the emergency medical services dispatch center^g,h^	Open
**3: prior BLS experience**		
	Current or past student of a health care profession, BLS instructor, or professional rescuer	MAQ^i^
	Prior BLS training	Yes/No
	Wish to be trained, or more trained, in BLS procedures	Yes/No
**4: specific BLS knowledge**		
	Criteria used to recognize OHCA^g,h,j^	MAQ
	BLS sequence^g,h^	Ordering
	Artery for pulse assessment^g^	MCQ
	Compression depth^g,h^	MCQ
	Compression:ventilation ratio^g^	MCQ
	Compression rate^g,h^	MCQ
	Compression-only CPR^g,h,k^	Yes/No
	Treatment of a choking patient, conscious, unable to either cough or talk^g^	MCQ
	Self-assessed confidence in the ability to perform resuscitation	1-10 scale

^a^N/A: not applicable.

^b^Regex: regular expression validation.

^c^MCQ: multiple choice question (only one answer accepted).

^d^BLS: basic life support.

^e^ACLS: advanced cardiovascular life support*.*

^f^AED: automated external defibrillator.

^g^Questions used to compute the primary outcome (score out of 10 questions).

^h^Questions used to compute the “essential BLS knowledge” secondary outcome.

^i^MAQ: multiple answer question (more than one answer accepted).

^j^OHCA: out-of-hospital cardiac arrest.

^k^CPR: cardiopulmonary resuscitation.

### Enrollment and Consent

**Figure 1:**
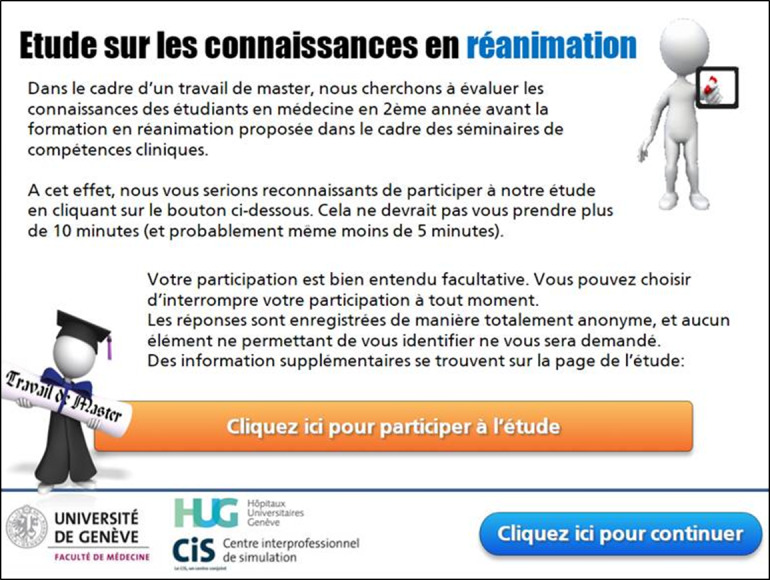
Electronic learning (eLearning) slide inviting medical students to participate in the study.

As the UGFM BLS-AED course is based on a “flipped classroom” teaching format, medical students must complete an institutional, interactive, electronic learning (eLearning) module prior to attending their first 2-hour BLS-AED workshop. A link was therefore embedded in the very first slide of the eLearning module (Figure 1), prompting the 183 second-year UGFM students, which represented a convenience sample, to participate in the study before accessing the learning material.

The control group was composed of lay people attending a first aid course. These participants were recruited thanks to the Association Genevoise des Sections de Samaritains (AGSS), a Red Cross–affiliated training center. The AGSS agreed to send a single mailing (Multimedia Appendix 2) on our behalf to each first aid course participant who registered between October 2019 and April 2020. In Switzerland, first aid courses are mandatory to obtain a driving license [18]. Though anonymity was ensured by virtue of the invitation method, no reminder could be sent as we did not have access to the participants’ email addresses or to their identities. These invitation emails were sent at least 2 weeks before these courses were scheduled, and participants were specifically asked to take the questionnaire before attending the course.

Information regarding the study was displayed along with an electronic consent form before the questionnaire could be started [19]. Participants were informed of the study’s purpose and of its estimated length. Identity and contact of the investigators were given, and information regarding data handling was provided. Participants were informed that no personal data would be asked for or recorded and that they would not be solicited any further after having completed the questionnaire.

No incentive was given to promote participation, which was not required to attend either course.

### Data Collection

All questions had to be answered, and pages were to be entirely filled before participants could proceed to the following part of the survey. Answer consistency was checked using regular expression (regex) validation rules, and participants were warned whenever an inconsistent answer was identified. Participants could change their answers using a “back” button until the questionnaire was finalized.

Data were stored on an encrypted MySQL compatible database (MariaDB 5.5.5, MariaDB Foundation, Wakefield, MA) located on a Swiss server. As this was a closed survey, with the link only provided either on the first eLearning slide or in the emails sent by the AGSS, no specific tracking identifier (cookies or IP address) was used.

### Outcomes

The primary outcome was the between-group difference on the 10-question quiz score. Each question was equally weighted and could only be considered as correct or incorrect. Thus, the total score was computed for each participant by summing all correct answers. Secondary outcomes were the differences in the rate of correct answers for each individual question and in the level of self-assessed confidence in the ability to perform resuscitation. We also computed a score dubbed “essential BLS knowledge,” which is the sum of 6 critical questions related to BLS (Table 1) and follows the logic of the chain of survival. Indeed, the other questions used to compute the primary outcome are either related to other first aid maneuvers or are not deemed of critical importance to the adequate provision of resuscitative maneuvers.

### Data Availability

The original data have been deposited to Mendeley Data [20].

### Statistical Analysis

Statistical analysis was carried out using STATA 16.1 (StataCorp LLC, College Station, TX). Incomplete questionnaires, as well as those completed by BLS-AED instructors or already certified health care professionals, were excluded from the analysis.

The scores of the answers to the 10 predefined questions were added to compute the overall quiz score defined as the primary outcome (minimum = 0; maximum = 10). No differential weighting was applied, and each individual question was worth 1 point. The “essential BLS knowledge” score was computed in the exact same way (minimum = 0; maximum = 6).

Normality was assessed both graphically and by the Kolmogorov-Smirnov test. Analysis of continuous variables was first performed using the Student *t* test, and results were then adjusted according to age, gender, and prior BLS experience through the use of multivariable linear regression. Results are presented as mean (SD). Given the limited sample size, the Fisher exact test was used for between-groups comparison of binary variables. A double-sided *P* value <.05 was considered significant.

A subgroup analysis to identify a potential effect of having attended a BLS course prior to taking the survey was decided post hoc. A sensitivity analysis excluding medical students who had prior training as health care students or who were already certified rescuers was also performed.

## Results

**Figure 2:**
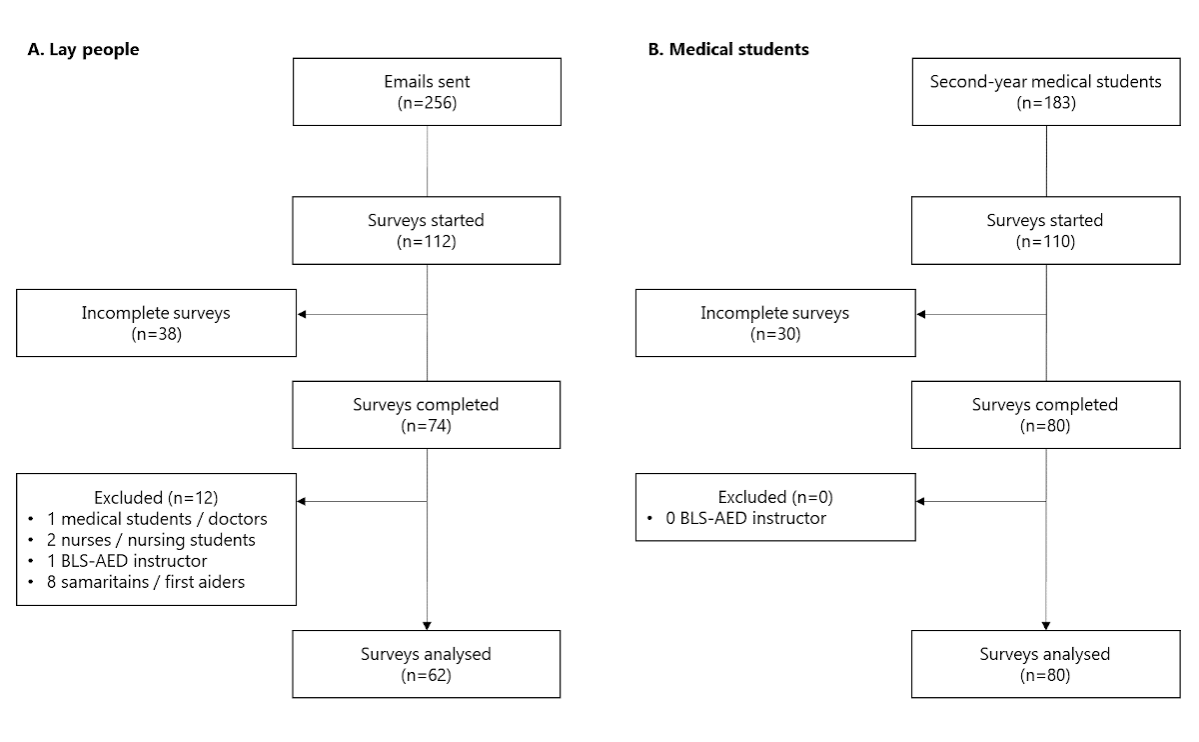
Study flowchart for (A) lay people and (B) medical students. AED: automated external defibrillator; BLS: basic life support.

The participation rate was higher (*P*=.03) in the medical students’ group (80/183, 44%) than in the control group (74/256, 29%). While no participant had to be excluded from the medical students’ group, the exclusion criteria were met for 12 participants in the control group (Figure 2). Among the 80 medical students who completed the survey, 1 was a former nursing student, and 13 answered they were certified rescuers. There were no fully qualified health care professionals in this group.

Participants were older in the control group (mean 34.0 years, SD 12.7 years) than in the medical students’ group (mean 22.5 years, SD 4.4 years). There was a majority of women in both groups (49/74, 66% in the control group and 58/80, 73% in the medical students’ group) with no significant difference between groups (*P*=.35). While the proportion of participants who had already heard of BLS or of advanced cardiovascular life support was also similar (48/80, 60% of medical students vs 41/74, 55% of lay people; *P*=.39), medical students more often declared having already attended a BLS course (68/80, 85% vs 45/74, 61%, *P*<.001).

The mean score on the 10-question composite outcome was higher in medical students (mean 5.8, SD 1.7 vs mean 4.2, SD 1.7; *P*<.001), with 6 questions displaying significant differences (Table 2). Older participants were more likely to score lower, with a coefficient of –0.031 per year (95% CI –0.060 to –0.003, *P*=.03), while the participants who had attended a BLS course prior to taking the questionnaire scored higher (1.166, 95% CI 0.470 to 1.862, *P*=.001). No participant was able to correctly answer all 10 questions.

Medical students also scored better than lay people on the 6-question “essential BLS knowledge” score (mean 3.0, SD 1.1 vs mean 2.0, SD 1.0; *P*<.001). Older age was also correlated with lower scores (coefficient –0.025 per year, 95% CI –0.043 to –0.007, *P*=.007), and participants who had attended a BLS course prior to completing the questionnaire also scored higher on this index (0.698, 95% CI 0.258 to 1.138, *P*=.002). Once again, no participant was able to correctly answer all 6 questions. Neither score was affected by gender.

Lay people who had already attended a BLS course before participating in the survey did not perform better than those who had not (mean 4.5, SD 1.6 vs mean 3.8, SD 1.7, *P*=.14). The same held true for medical students (mean 5.9, SD 2.2 vs mean 5.4, SD 1.6, *P*=.39).

Medical students felt more confident than lay people in their ability to perform resuscitation (mean 4.7, SD 2.2 vs mean 3.1, SD 2.1, *P*<.001). Participants who had already attended a BLS course felt more confident than those who had not (coefficient 1.9, 95% CI 1.1 to 2.7, *P*<.001), while female participants felt less confident (coefficient –1.1, 95% CI –1.9 to –0.4, *P*=.003). Older participants felt less confident than their younger counterparts (coefficient –0.041 per year, 95% CI –0.074 to –0.009, *P*=.01). The proportion of participants wishing to receive more BLS training was higher in the group composed of medical students (70/80, 88% vs 42/74, 57%, *P*<.001).

Excluding medical students who were either former nursing students or certified rescuers neutralized the effect of age on the 10-question score and on the confidence but did not significantly change the magnitude or direction of the other results.

**Table 2:** Scores on the 10-question composite outcome by group.

Question	Lay people (n=62), n (%)	Medical students (n=80), n (%)	*P* value
Phone number of the emergency medical services dispatch central^a^	51 (82)	75 (94)	.06
Meaning of “AED”^b,c^	27 (44)	42 (53)	.31
Criteria used to recognize OHCA^d^	5 (8)	16 (20)	.06
BLS^e^ sequence	2 (3)	0 (0)	.19
Artery for pulse assessment	50 (80)	50 (63)	.03
Compression depth	9 (14)	29 (36)	.004
Compression:ventilation ratio	21 (34)	62 (78)	<.001
Compression rate	15 (24)	41 (51)	.002
Compression-only CPR^f^	39 (63)	75 (94)	<.001
Treatment of a choking patient, conscious, unable to either cough or talk	43 (69)	75 (94)	<.001

^a^Accepted answers: 112, 144, and 911, which all work in Geneva, Switzerland; 144 is the official Swiss number.

^b^AED: automated external defibrillator.

^c^All answers containing “defibrillator” in English or in French were considered as correct (not case sensitive, spelling mistakes accepted).

^d^OHCA: out-of-hospital cardiac arrest.

^e^BLS: basic life support.

^f^CPR: cardiopulmonary resuscitation.

## Discussion

### Principal Findings

In this study, second-year medical students performed somewhat better than lay people, though neither group scored high on a simple 10-question quiz assessing BLS knowledge. The difference might arise from different grounds. First of all, medical students might indeed be more interested in this domain given their chosen profession. Moreover, though our regional policies have evolved little with regard to BLS training promotion, private initiatives have progressively surfaced in an attempt to increase general awareness regarding OHCA. As medical students were more than a decade younger than the control group, they might have been more exposed to such campaigns and therefore more interested in this topic.

Our findings highlight weaknesses in the first 3 links of the chain of survival [21]. While the proportion of participants who correctly remembered the phone number of the medical emergency communication center was high in both groups, most were unable to correctly describe the criteria used to identify OHCA. Failure to correctly identify OHCA may lead to a delayed call to the dispatch center and thereby worsen the victim’s prognosis [22]. Once called, dispatchers should nevertheless be able to help identify OHCA and lead the bystander to start appropriate actions [23]. However, while cardiopulmonary resuscitation (CPR) might be initiated, our survey results suggest that compression could be of limited quality since answers related to compression rate and depth were mostly incorrect. These findings are worrying since high-quality chest compressions are of paramount importance and have been shown to improve survival outcomes [24-26]. The third link in the chain, defibrillation, was only assessed with one simple question that focused on the meaning of the AED abbreviation. Less than half of all participants were able to determine that these 3 letters were commonly used to refer to a defibrillator. However, one could reasonably expect that associating the lightning pictograph to the abbreviation might improve identification of these devices [27]. Moreover, most emergency medical dispatchers are now trained to help bystanders or first responders localize, retrieve, and use AEDs [28].

Although lay rescuers, either trained or untrained, have not been expected to check for a pulse since the publication of the 2010 CPR guidelines [29], we nevertheless elected to ask a question regarding pulse assessment. Indeed, our BLS teaching faculty considers even junior medical students to be health care providers, and these students are therefore expected to know how to check for a pulse, particularly to assess a potential return of spontaneous circulation [30]. It must however be unequivocal that the pulse check should not be performed to diagnose cardiac arrest as it has been shown to be highly unreliable [31].

Even though emergency medical systems have evolved to overcome as much as possible the lack of BLS training [32], developing first aid knowledge and skills among the general population is essential to improve outcomes not only in OHCA victims but also in patients presenting with other acute illnesses or injuries [33]. Many different strategies can be considered to enhance the global level of awareness regarding OHCA and CPR, including systematic teaching of BLS maneuvers to school children [34-36]. This strategy has been shown to be particularly effective, as children are eager to transfer their acquired knowledge to their parents, siblings, and friends [37,38]. Training school children and providing them with cheap, basic manikins has been shown to help disseminate CPR training among their relatives [39], and many European countries have adhered to the “KIDS SAVE LIVES” statement, which aims to provide BLS education to schoolchildren aged 12 years and older [40]. Until such a change in BLS education policies occurs, medical students should be offered first aid courses earlier in their curriculum. The recently released European Resuscitation Council guidance note related to the CPR competencies required in undergraduate health care students could be used as a guideline to facilitate the implementation of BLS courses sooner in the undergraduate curriculum [41]. This note advocates, as we do, for the mandatory teaching of BLS maneuvers already during the first year of undergraduate education for students of health care professions.

However, months, if not years, might elapse before any change in public health policies can be accomplished, and modifying the undergraduate medical curriculum might take just as long. Therefore, to enhance the awareness of UGFM junior medical students regarding BLS issues, a team of senior medical students and faculty members developed an accelerated first aid course [42]. In January 2021, first-year medical students attended a brief intervention inviting them to complete an eLearning module. In the context of the current COVID-19 pandemic, asynchronous distance learning using interactive eLearning modules has been shown to enhance knowledge acquisition in medical students [43]. After completing this module, first-year medical students were invited to register for 1-hour practice sessions. Students who successfully complete this learning path will be able to enlist as first responders. Future studies should determine whether this strategy is successful and can improve BLS knowledge in this population.

The results of our post hoc analysis regarding the effect of having attended a BLS course prior to taking the survey are cause for concern. Indeed, participants who declared having attended such a course in the past did not perform significantly better than those who did not, regardless of the study group. While a change in the guidelines between a prior course and the moment of the survey could be hypothesized, the young age of the participants, particularly in the medical students’ group, makes it unlikely. Low scores were indeed recorded regarding key elements that were already part of the 2010 guidelines (ie, the criteria used to recognize OHCA [29]). Many factors, such as the time elapsed since the last BLS course, the course format, or the number of previous courses the participants had attended, may explain the apparent lack of BLS knowledge retention [44-46]. However, as this was an unexpected finding, the design of our study was ill suited to explore its grounds, and further studies will be needed to confirm this result and try to determine potential causes.

This study has other limitations that should also be acknowledged. First, owing to the study design and to the impossibility of sending email reminders, the participation rate was rather low, particularly among lay rescuers. This might have led to an overestimation of BLS knowledge in both groups due to selection bias. The method of recruitment has been shown to significantly alter the participation rates [47,48], and previous studies have reported that paper questionnaires have slightly higher response rates than web-based ones [49]. The use of paper questionnaires is however associated with much higher costs and carries an increased risk of generating missing values.

In addition, we were unable to determine whether the questionnaire had actually been completed before the course. Nevertheless, medical students were required to complete the eLearning module with the embedded invitation slide before attending their first BLS course, and lay people were sent the invitation email at least 2 weeks before attending their course.

Another limitation is that our control group cannot be considered as a true surrogate of the general population. Indeed, as participants were rather young and as BLS training initiatives have progressively increased, it is to be expected that BLS knowledge would actually be lower in a more representative sample of the general population. Another limitation is linked to the specificities of the UGFM curriculum. In our curriculum, medical students and dental medicine students share a common study path until the end of their second year of undergraduate training. Around one-fifth of second-year UGFM students are actually dental medicine students. There is however little reason to believe that their interest in BLS procedures should be different for these students compared to medical students whose interest is not in an acute medicine specialty. In addition, most junior medical students have not yet decided upon a specific career at this stage [50]. One last limitation is that no survey can be considered as a true assessment of the way bystanders would manage OHCA. Nevertheless, there is a correlation between confidence, CPR skills, and intention to perform resuscitation, and a low level of knowledge can hardly foster confidence [51].

### Conclusion

Although medical students were more knowledgeable than lay people regarding BLS-AED procedures, their proportion of correct answers was still low. As OHCA recognition and high-quality chest compressions are paramount to increasing survival rates, a change in the curriculum, as well as a global transformation in the way the general population is educated regarding first aid maneuvers, could help improve outcomes.

## Appendix

Multimedia Appendix 1 Questions and expected answers: original (French) version and English translation.

Multimedia Appendix 2 Text sent in the invitation e-mails (dispatched on our behalf by the Association Genevoise des Sections de Samaritains).

## Abbreviations

AED: automated external defibrillator
AGSS: Association Genevoise des Sections de Samaritains
BLS: basic life support
CPR: cardiopulmonary resuscitation
eLearning: electronic learning
OHCA: out-of-hospital cardiac arrest
UGFM: University of Geneva Faculty of Medicine
