# Supplementary material for: Differences in Basic Life Support Knowledge Between Junior Medical Students and Lay People: Web-Based Questionnaire Study
Source: J Med Internet Res. 2021 Feb 23;23(2):e25125. doi: 10.2196/25125 (PMC7943337; doi:10.2196/25125)
Supplement: Multimedia Appendix 1 [file jmir_v23i2e25125_app1.pdf]

Supplementary Table 1

Questions and expected answers: original (French) version and English translation

#	Questions and answers (FR)	Questions and answers (EN)
1	***Quel est le numéro à appeler en cas d'urgence médicale ?***<br><br>*Réponses acceptées (texte libre) :* 144, 112, 911	***What is the medical emergency number?***<br><br>*Accepted answers (free text):* 144, 112, 911
2	***Quels sont les critères qui doivent être présents pour affirmer qu'un patient est en ACR ?***<br><br>*Question à réponses multiples (≥ 1 réponse possible):*<br>☑ Absence de réponse à la stimulation verbale et tactile<br>☑ Respiration absente ou anormale (lente et laborieuse)<br>☐ Absence de pouls central<br>☐ Langage désorganisé (ou incohérent)<br>☐ Absence de pouls périphérique	***Among the following, which criteria should be used to recognize cardiac arrest?***<br><br>*Multiple answer question (≥ 1 possible answer):*<br>☑ No reaction to verbal or tactile stimuli<br>☑ No or abnormal breathing (slow and labored)<br>☐ Absence of a central pulse<br>☐ Disorganized (or confused) speech<br>☐ Absence of a peripheral pulse
3	***Pour traiter un patient en arrêt cardiorespiratoire, dans quel ordre procédez-vous ?***<br><br>*Mettre les propositions dans l'ordre:*<br>1. Compressions thoraciques<br>2. Libération des voies aériennes<br>3. Insufflations	***In what order do you proceed to treat a patient in cardiopulmonary arrest?***<br><br>*Ordering:*<br>1. Chest compressions<br>2. Opening the airway<br>3. Rescue breaths
4	***Quelle est l'artère idéale pour ressentir un pouls chez un patient adulte ?***<br><br>Question à choix multiple (une seule réponse):<br>☐ L'artère radiale<br>☑ L'artère carotide<br>☐ L'artère brachiale<br>☐ L'artère fémorale	***What is the ideal artery for feeling a pulse in an adult patient?***<br><br>*Multiple choice question (only one possible answer):*<br>☐ The radial artery<br>☑ The carotid artery<br>☐ The brachial artery<br>☐ The femoral artery
5	***A quelle profondeur la cage thoracique doit-elle être enfoncée chez un adulte ?***<br><br>Question à choix multiple (une seule réponse):<br>☐ 2-3 cm<br>☐ 3-4 cm<br>☐ 4-5 cm<br>☑ 5-6 cm<br>☐ 6-7 cm	***How deep should the chest compressions be in an adult victim?***<br><br>*Multiple choice question (only one possible answer):*<br>☐ 2-3 cm<br>☐ 3-4 cm<br>☐ 4-5 cm<br>☑ 5-6 cm<br>☐ 6-7 cm
6	***Quel est le ratio compressions / insufflations à effectuer ?***<br><br>Question à choix multiple (une seule réponse):<br>☐ 15:2<br>☐ 25:2<br>☐ 30:1<br>☑ 30:2<br>☐ 60:4	***What compression/ventilation ratio should you use in case of cardiac arrest?***<br><br>*Multiple choice question (only one possible answer):*<br>☐ 15:2<br>☐ 25:2<br>☐ 30:1<br>☑ 30:2<br>☐ 60:4
7	***A quelle fréquence les compressions doivent-elles être effectuées ?***<br><br>Question à choix multiple (une seule réponse):<br>☐ 60-80 compressions par minute<br>☐ 80-100 compressions par minute<br>☑ 100-120 compressions par minute<br>☐ 120-140 compressions par minute<br>☐ 140-160 compressions par minute	***Which chest compression rate should you use?***<br><br>*Multiple choice question (only one possible answer):*<br>☐ 60-80 compressions per minute<br>☐ 80-100 compressions per minute<br>☑ 100-120 compressions per minute<br>☐ 120-140 compressions per minute<br>☐ 140-160 compressions per minute

#	Questions and answers (FR)	Questions and answers (EN)
8	***Si aucune insufflation n'est administrée, les compressions thoraciques restent-elles utiles ?***<br><br>*Question à choix multiple (une seule réponse):*<br>☑ Oui<br>☐ Non<br>☐ Je ne sais pas	***If rescue breaths are not performed, are chest compressions still helpful?***<br><br>*Multiple choice question (only one possible answer):*<br>☑ Yes<br>☐ No<br>☐ I don't know
9	***Quelle est la première action recommandée face à un patient adulte qui s'étouffe, sachant qu'il est incapable de parler ni de tousser ?***<br><br>Question à choix multiple (une seule réponse):<br>☑ Tenter une manœuvre de Heimlich<br>☐ Commencer une réanimation cardio-pulmonaire<br>☐ Maintenir le patient en position de Trendelenburg<br>☐ Aller chercher l'obstacle dans la bouche du patient	What is the recommended first action when faced with an adult patient who is choking and unable to speak or cough?<br><br>*Multiple choice question (only one possible answer):*<br>☑ A Heimlich maneuver<br>☐ Initiating cardiopulmonary resuscitation<br>☐ Keep the patient in a Trendelenburg position<br>☐ Do a finger sweep to remove the obstacle
10	***Que veut dire DSA ?***<br><br>Réponses acceptées (texte libre) : toute réponse contenant le mot « défibrillateur », sans tenir compte de la casse ni	***What does DSA mean?***<br><br>Answers accepted (free text): all answers containing the word « defibrillator », regardless of how it was spelled.

#, question number
