# Supplementary material for: Differences in Basic Life Support Knowledge Between Junior Medical Students and Lay People: Web-Based Questionnaire Study
Source: J Med Internet Res. 2021 Feb 23;23(2):e25125. doi: 10.2196/25125 (PMC7943337; doi:10.2196/25125)
Supplement: Multimedia Appendix 2 [file jmir_v23i2e25125_app2.pdf]

Madame, Monsieur,

Vous recevez ce courriel car vous vous êtes récemment inscrit.e à un cours de premiers secours BLS (Basic Life Support).

Je m’appelle Ludovic Sturny et je suis étudiant en 5ème année de médecine à l’Université de Genève. Dans le cadre de mon travail de master, vous êtes cordialement invité.e à répondre à un questionnaire en ligne avant de recevoir votre formation.

En voici le lien : https://etude-bls.cardiomobile.ch/agss

Le but de cette étude est d’analyser les connaissances préalables en BLS de la population générale.

Cinq minutes sont suffisantes pour répondre à la quinzaine de questions qui portent sur les connaissances générales en matière de réanimation cardio-respiratoire. Le questionnaire peut aisément être complété via smartphone.
Comme vous pourrez le constater dès la première page du questionnaire, nous vous assurons que vos données sont totalement anonymes.

En vous remerciant d’avance pour l’attention que vous porterez à ma démarche et votre collaboration, je vous prie d’agréer, Madame, Monsieur, mes salutations distinguées.
